# A Neuron Projection Guidance Signature Associates with Immune-Cold Tumor Microenvironment and Poor Outcomes in Breast Cancer

**DOI:** 10.1245/s10434-026-19932-3

**Published:** 2026-07-25

**Authors:** Kei Kawashima, Kohei Chida, Masanori Oshi, Akimitsu Yamada, Kazutaka Narui, Shipra Gandhi, John M. L. Ebos, Scott I. Abrams, Arin Bhattacharjee, Itaru Endo, Kazuaki Takabe

**Affiliations:** 1 Department of Surgical Oncology, Roswell Park Comprehensive Cancer Center, Buffalo, NY USA; 2 Department of Gastroenterological Surgery, Yokohama City University Graduate School of Medicine, Kanagawa, Japan; 3 Department of Breast and Thyroid Surgery, Yokohama City University Medical Center, Yokohama City, Kanagawa Japan; 4 Department of Medicine, Winship Cancer Institute of Emory University, Atlanta, GA USA; 5 Department of Cancer Genetics and Genomics, Roswell Park Comprehensive Cancer Center, Buffalo, NY USA; 6 Department of Immunology, Roswell Park Comprehensive Cancer Center, Buffalo, NY USA; 7 Department of Pharmacology and Toxicology, University at Buffalo Jacobs School of Medicine and Biomedical Sciences, The State University of New York, Buffalo, NY USA; 8 Department of Surgery, University at Buffalo Jacobs School of Medicine and Biomedical Sciences, The State University of New York, Buffalo, NY USA; 9 Division of Digestive and General Surgery, Niigata University Graduate School of Medical and Dental Sciences, Niigata, Japan; 10 Department of Breast Surgery and Oncology, Tokyo Medical University, Tokyo, Japan; 11 Department of Breast Surgery, Fukushima Medical University, Fukushima, Japan

**Keywords:** Triple-negative breast cancer, Intratumoral nerve, Axonogenesis, Tumor microenvironment, Treatment response, Transcriptome

## Abstract

**Background:**

Neural involvement in the tumor microenvironment (TME) has increasingly been recognized as a regulator of cancer aggressiveness. However, its characterization in breast cancer remains a challenge due to very low nerve density in this cancer type. This study investigates the clinical relevance of cancer-medicated nerve recruitment activity in the TME of breast cancer patients.

**Methods:**

Transcriptomic and clinical data from 9017 breast cancer patients across multiple cohorts were analyzed. A Neuron Projection Guidance (NPG) score generated by GSVA of a MSigDB gene set was used to quantify nerve recruitment activity. Patients were dichotomized by cohort-specific medians.

**Results:**

The NPG score was associated with axonogenesis-related gene expressions. NPG-high tumors exhibited lower cell proliferation, indicated by less Ki67 gene expression, and less enrichment of proliferation-related Hallmark gene sets. NPG-high tumors revealed significantly lower mutational burdens and neoantigen loads, which were consistent with an immune-cold TME, characterized by lower infiltration of immune effector cells, decreased cytolytic activity, and suppressed immune-related pathways. NPG scores were higher in patients who did not achieve pathological complete response after neoadjuvant chemotherapy. Moreover, high NPG scores were associated with poorer survival. These associations were observed in triple-negative breast cancer (TNBC), but not consistently in ER+/HER2− nor HER2+ subtypes.

**Conclusions:**

These findings reveal a previously unrecognized tumor-neuronal cross-talk in TNBC biology, which associates with an immune-cold TME, reduced chemotherapeutic efficacy, and unfavorable clinical outcomes. Such a network has potential implications for risk stratification and therapeutic decision-making in TNBC.

**Supplementary Information:**

The online version contains supplementary material available at https://doi.org/10.1245/s10434-026-19932-3.

Intratumoral nerves, the nerves inside a bulk tumor, are now increasingly recognized as an important regulator of tumor biology,^1^ with many solid cancers containing sensory and autonomic peripheral nerve fibers within the tumor microenvironment (TME).^2,3^ This phenomenon is prevalent in cancers arising from highly innervated organs, such as pancreatic and gastric cancer, but also occurs in many other cancers that originate in tissues where nerves are sparse, including breast cancer. This fundamental discovery reflects not only the abundance of pre-existing nerves within the original tissue but also the active recruitment of new neural projections toward the TME, a cancer-mediated process known as axonogenesis.^4,5^ Recruitment of these neural projections is stimulated by neurotrophic factors released by cancer cells and can be directed by axon-guidance molecules in the TME.^6^ Consequently, many cancers display significantly higher intratumoral nerve density compared with adjacent normal tissue.^7^ Tumors with aggressive phenotypes, such as those with higher histological grade and poorer differentiation, have been shown to contain higher densities of intratumoral innervation,^5,8,9^ suggesting that neural projection recruitment is more active in highly malignant tumors. Indeed, increased nerve density within the TME is associated with poorer prognoses across many cancers, such as pancreatic cancer, gastric cancer, and prostate cancer.^10,11^

In breast cancer, intratumoral nerves are identified in about 30–80% of cases.^10,12^ Higher nerve density has been linked to increased cancer cell proliferation, lymph node metastases, and recurrences.^12–14^ However, the density of nerve fibers within breast tumors is extremely low, reported to cover only around 2% of the tumor area,^15^ making them challenging to detect and to further elucidate their functions within the TME.^10^ Interestingly, triple-negative breast cancer (TNBC), which is the most aggressive subtype among breast cancers, shows elevated expression of neurotrophic factors and axon-guidance pathways,^16^ even though histological detection of physical nerve fibers can vary significantly.^10,12^ In addition, TNBC is the subtype of breast cancer associated with potentially the highest sympathetic activity as demonstrated by improved survival with beta-blockers in TNBC,^17–19^ compared with other subtypes of breast cancer. While TNBC is often regarded as a highly proliferative and chemotherapy-responsive subtype, clinical responses, and survival outcomes are highly variable, indicating underlying biological heterogeneity within TNBC. These observations imply that signaling related to neural projection recruitment better reflect nerve abundance within the TME compared with conventional histological analyses, with particular relevance to TNBC, thereby forming the rationale for our main focus on TNBC in this study.

In this study, we sought to investigate the clinical relevance of cancer-mediated induction of neural projections toward the TNBC TME. To do this, we used the Gene Set Variation Analysis (GSVA), which is a computational algorithm that utilizes coordinated gene expression levels as a score to characterize pathway activity instead of focusing on individual genes.^20^ Our group has successfully applied GSVA to study pathway activities in multiple types of cancer, including the Hippo pathway,^21^ angiogenesis,^22^ and reactive oxygen species^23^ in breast cancer, glycolysis in hepatocellular carcinoma,^24^ and the G2M checkpoint in pancreatic cancer.^25,26^ Using multiple databases containing transcriptomic data, we generated a Neuron Projection Guidance (NPG) score to assess whether NPG activity is associated with key cancer hallmarks within the TME, including immune activity and cell proliferation. Furthermore, we investigated how NPG activity relates to response to chemotherapy and survival outcomes. Together, this study connects tumor-driven neural projection guidance activity with immune-cold biology, reduced chemotherapy response, and poor clinical outcomes in TNBC.

## Material and Methods

### Data Acquisition

Clinical and transcriptomic data from a total of 9,017 patients with breast cancer were analyzed. Primary analyses, including clinical outcome, immune infiltration profiling, genomic feature characterization, and pathway enrichment, were conducted using three large independent breast cancer cohorts: The Cancer Genome Atlas (TCGA; n = 1,081 overall, n = 170 TNBC), the Molecular Taxonomy of Breast Cancer International Consortium (METABRIC; n = 1,980 overall, n = 335 TNBC), and the Sweden Cancerome Analysis Network-Breast (SCAN-B; n = 3,409 overall, n = 174 TNBC). To investigate the association between the NPG score and response to neoadjuvant chemotherapy (NAC), we analyzed seven independent cohorts that underwent NAC: Wolf et al. (ISPY-2 trial; GSE194040; n = 987 overall, n = 363 TNBC)^27^; Hatzis et al. (GSE25066; n = 327 overall, n = 133 TNBC)^28^; Chen et al. (GSE163882; n = 222 overall, n = 90 TNBC)^29^; Ding et al. (GSE123845; n = 222 overall, n = 86 TNBC)^30^; Pusztai et al. (GSE20271; n = 178 overall, n = 63 TNBC)^31^; Sota et al. (GSE230881; n = 263 overall, n = 49 TNBC)^32^; and Horak et al. (GSE41998; n = 279 overall, n = 149 TNBC).^33^ Clinical and transcriptomic data of TCGA and METABRIC were downloaded from cBioPortal and UCSC Xena as described previously.^34–38^ The same analytical framework was additionally applied to ER+/HER2− and HER2+ breast cancers across all cohorts, and the corresponding results are provided in the Supplementary Figs. S1–S5. Data for all other cohorts were obtained from the Gene Expression Omnibus (GEO) by using the GEOquery package in R. All datasets were downloaded in October 2025. Because all data used in this study were derived from publicly available, de-identified sources, institutional review board (IRB) approval was not required.

### Generation of the Neuron Projection Guidance (NPG) Score

A gene set comprising 244 genes annotated under the Gene Ontology term “neuron projection guidance” was obtained from the Molecular Signatures Database (MSigDB; Table S1). The Neuron Projection Guidance (NPG) score was defined as the GSVA-derived enrichment score of this gene set. Patients were stratified into NPG-high and NPG-low groups using the median NPG score within each cohort as the cutoff.

### Cell Composition, Cytolytic Activity Score, and Immune-Related Scores

To estimate the infiltration levels of immune cell populations within the TME, we applied the xCell algorithm.^39^ The cytolytic activity (CYT) score was calculated according to the method described by Rooney et al.,^40^ defined as the geometric mean of granzyme A (GZMA) and perforin (PRF1) expression. For the assessment of tumor immunogenicity and proliferative activity in the TCGA cohort, we used the precomputed scores reported by Thorsson et al.^41^

### Gene Set Enrichment Analysis

Differentially expressed genes (DEGs) were identified by using the limma package. Subsequently, functional enrichment analysis was performed by using gene set enrichment analysis (GSEA) with the fgsea package, based on the Hallmark gene set collection from the MSigDB.^42^ The GSEA was conducted in a pre-ranked manner using log2 fold-change values for ranking genes. Statistical significance was defined as FDR < 0.25, and enrichment strength was evaluated by using the normalized enrichment score (NES).

### Association Between NPG Score and Response to Neoadjuvant Chemotherapy

To evaluate the association between the NPG score and response to neoadjuvant chemotherapy (NAC), we analyzed seven independent TNBC cohorts treated with anthracycline- and taxane-based NAC. Among these cohorts, only the I-SPY2 cohort (Wolf et al.; GSE194040) included treatment regimens with immune checkpoint inhibitors (ICI). Pathological complete response (pCR) status was extracted from the original clinical annotations of each cohort, as defined in the original studies. For the I-SPY2 cohort, analyses were performed separately for the overall cohort, including all treatment regimens and for the subset of patients treated with ICI- containing regimens. Within each cohort, NPG scores were calculated as described above and compared between patients who achieved pCR and those with residual disease (non-pCR). Statistical analyses were conducted independently within each cohort to account for differences in study design, treatment regimens, and transcriptomic platforms.

### Statistical Analysis

All statistical analyses and data visualization were performed by using R software (version 4.5.1) with Bioconductor (version 3.21). Group comparisons were performed by using the Kruskal-Wallis test, Mann-Whitney *U* test, or Fisher’s exact test, as appropriate. Survival analyses were conducted by using Kaplan-Meier estimation with differences assessed by the log-rank test. A *p*-value < 0.05 was considered statistically significant for all analyses.

## Results

### Neuron Projection Guidance Score Significantly Correlates with Neuron-Related Features

**Fig. 1:**
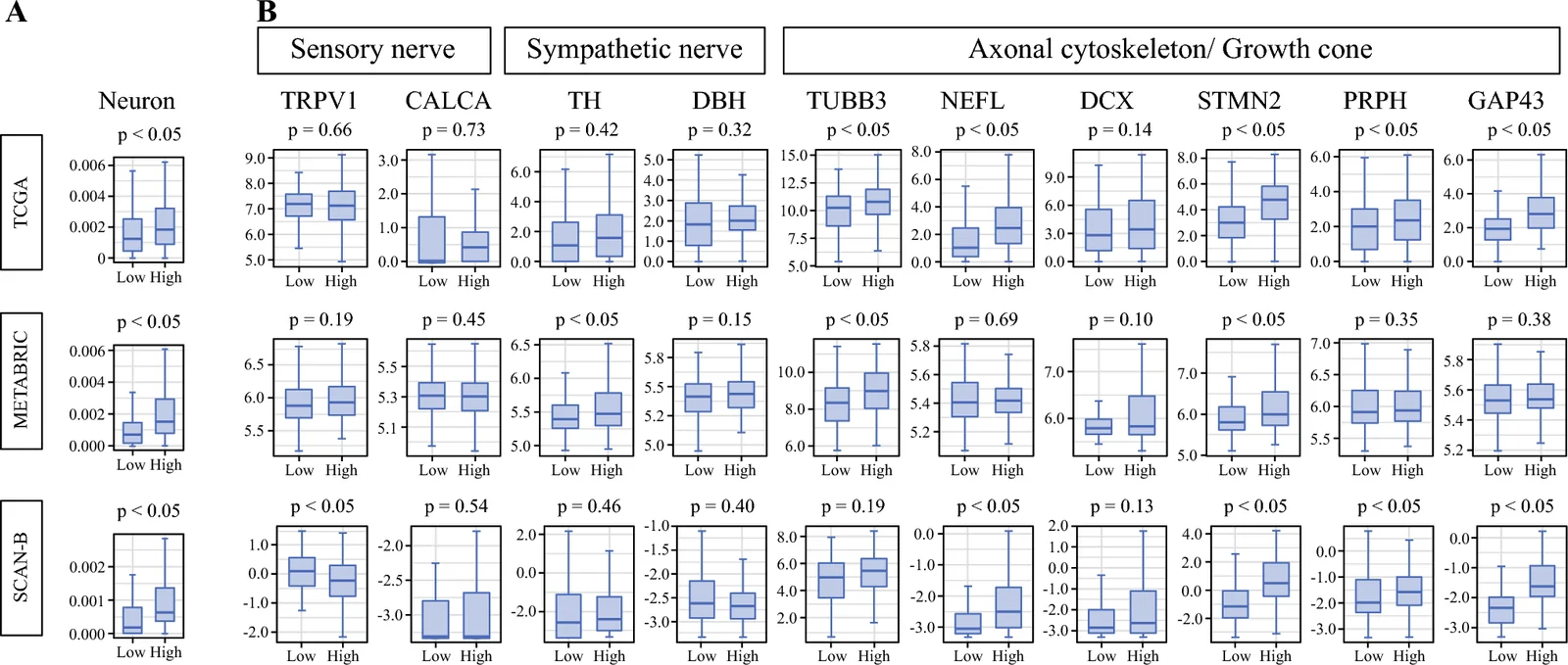
Association of the NPG score with neuron-related xCell score and gene expression. Box plots of NPG-high versus NPG-low TNBC patients in TCGA, METABRIC and SCAN-B cohorts showing (**A**) xCell Neuron score and (**B**) neuron-related gene expression. In the boxplots, the center line indicates the median, the box represents the interquartile range (IQR), and whiskers extend to 1.5×IQR. Outliers were omitted. *P* values were calculated by using the Wilcoxon rank-sum test. *TUBB3* tubulin beta 3; *NEFL* neurofilament light chain; *DCX* doublecortin; *STMN2* stathmin-2; *PRPH* peripherin; *GAP43* growth-associated protein 43; *TRPV1* transient receptor potential vanilloid 1; *CALCA* calcitonin related polypeptide alpha; *TH* tyrosine hydroxylase; *DBH* dopamine beta hydroxylase

Using the Molecular Signatures Database (MSigDB), we used 244 genes previously characterized as related to “neuron projection guidance” (NPG) to stratify multidatabase transcriptomic information obtained from TNBC patients. The patients were stratified into NPG-high and NPG-low groups by the median NPG score within each cohort. To validate that the NPG score reflects the induction of axonogenesis, we first investigated the extent to which the NPG score correlates with a neuron score and neuron-related marker gene expression levels. A neuron score, determined by the xCell algorithm, was found to be significantly higher in the NPG-high group across all three cohorts (all *p *< 0.05; Fig. 1A). Among classical neuronal marker genes, expression levels of sensory neuron markers (TRPV1, transient receptor potential vanilloid 1; CALCA, calcitonin-related polypeptide alpha) and sympathetic neuron markers (TH, tyrosine hydroxylase; DBH, dopamine beta-hydroxylase) did not show consistent associations with the NPG-high group across cohorts. In contrast, genes known to be enriched in axonal cytoskeleton and growth cones, such as TUBB3 (tubulin beta 3), NEFL (neurofilament light chain), STMN2 (stathmin-2), PRPH (peripherin), and GAP43 (growth-associated protein 43), were consistently elevated in NPG-high tumors (Fig. 1B). These results indicate that the NPG score captures axonogenesis-related activity within the TME, rather than simply reflecting the presence of mature nerve fibers. The NPG score distribution did not significantly differ among breast cancer subtypes (ER+/HER2−, HER2+, and TNBC) across all three cohorts (all *p *> 0.05; Supplementary Fig. S1A). Furthermore, similar patterns were observed in ER+/HER2− and HER2+ subtypes, where NPG-high tumors showed stronger associations with axonal cytoskeleton and growth cone–related genes than with classical markers of mature nerve fibers (Supplementary Fig. S1B).

### NPG Score Negatively Correlates with Tumor Proliferative Activity

**Fig. 2:**
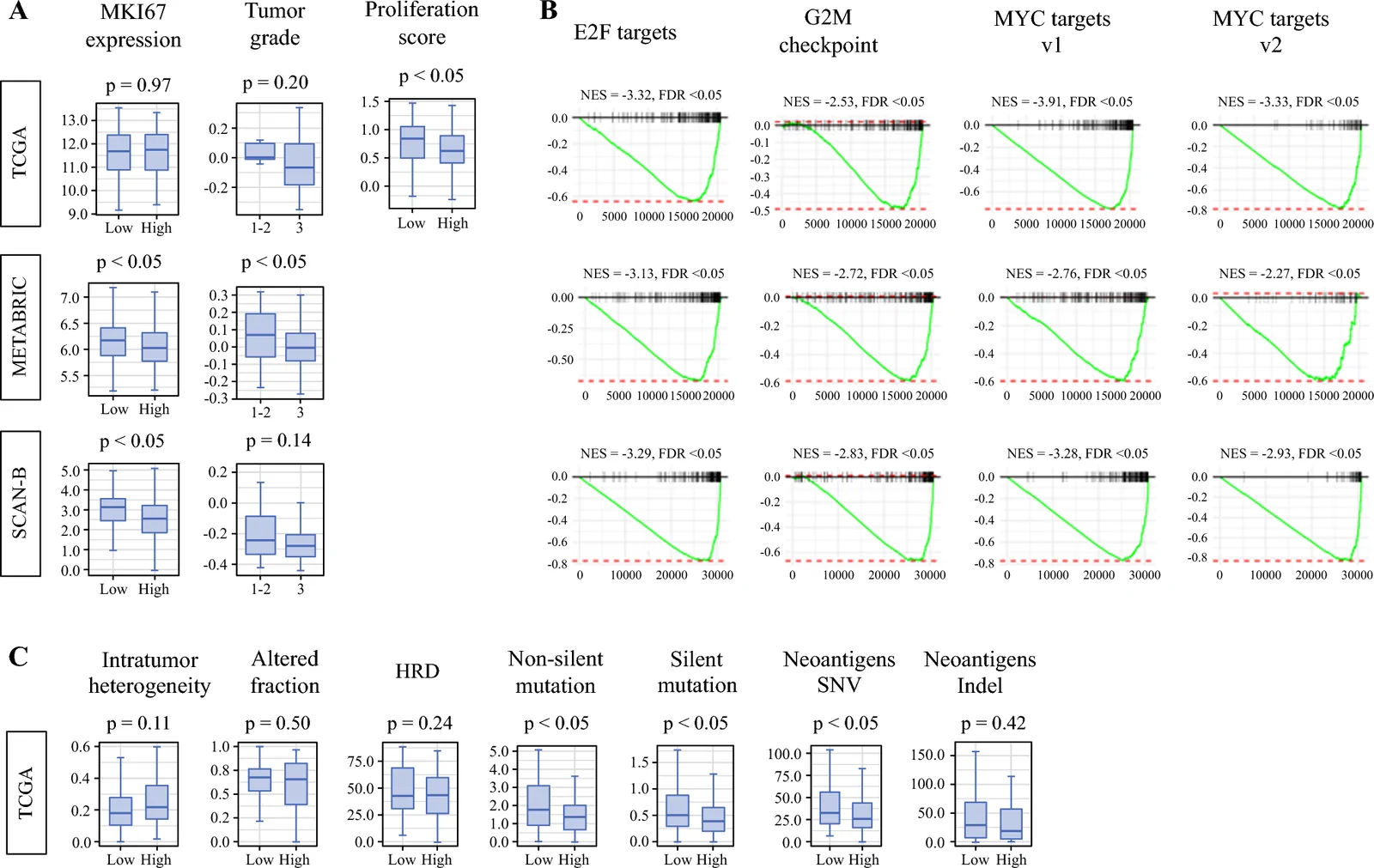
Association of NPG score and proliferative activity and tumor immunogenicity -related scores across TCGA, METABRIC, and SCAN-B cohorts. **A** Box plots showing MKI67 expression between NPG score-high and NPG score-low patients (left), a box plot of NPG score by tumor grade (middle), and a box plot of proliferation score between NPG score-high and NPG score-low patients (right). **B** Gene set enrichment analysis (GSEA) enrichment plots for proliferation-related Hallmark gene sets (E2F targets, G2M checkpoint, and MYC targets v1/v2) comparing NPG-high and NPG-low tumors in each cohort. The x-axis represents ranked genes ordered by differential expression between NPG-high and NPG-low groups, and the y-axis shows the enrichment score. Normalized enrichment score (NES) and false discovery rate (FDR) are shown for each pathway. **C** Boxplots of estimated scores of intratumor heterogeneity, altered fraction, homologous recombination deficiency (HRD), nonsilent and silent mutation burdens, single nucleotide variant (SNV)-derived neoantigens, and indel-derived neoantigens between NPG-high and NPG-low patients in TCGA cohort

Given that neuronal density has been associated with tumor aggressiveness, it was of interest to investigate the association between the NPG score and tumor proliferative activity. Interestingly, gene expression of Ki67, a molecular marker of cell proliferation, was lower in NPG-high tumors, although statistical significance varied across cohorts (TCGA: *p* = 0.97; METABRIC: *p* < 0.05; SCAN-B: *p* < 0.05; Fig. 2A left). Among pathological features, tumors with higher histological grade (grade 3) tended to have lower NPG scores compared with grade 1–2 tumors; however, this trend reached statistical significance in only one cohort (TCGA: *p* = 0.20; METABRIC: *p* < 0.05; SCAN-B: *p* = 0.14; Fig. 2A middle). Consistently, the proliferation score was significantly lower in the NPG-high group in the TCGA cohort (*p* < 0.05; Fig. 2A right). GSEA further supported these findings. Cell proliferation-related Hallmark gene sets, including MYC targets v1, MYC targets v2, E2F targets, and G2M checkpoint, were all enriched in the NPG-low group (all FDR < 0.05; Fig. 2B). These results indicate that TNBC with a high NPG score is less proliferative. Similar trends were observed in ER+/HER2− and HER2+ subtypes (Supplementary Fig. 2A).

### NPG Score is Associated with Lower Tumor Genomic Instability

Aggressive cancer cells are often linked to genomic instability; thus, it was of interest to investigate the relationship between NPG score and genomic instability in the TCGA cohort (Fig. 2C). Chromosomal instability, including intratumoral genomic heterogeneity, fraction-altered, and homologous recombination deficiency (HRD), showed no significant associations with the NPG score (all *p* > 0.05). In contrast, mutational burden-related factors, including nonsilent mutation rates, silent mutation rates, and SNV-derived neoantigen counts, were significantly lower in the NPG-high group (all *p* < 0.05). This pattern indicates that, despite comparable levels of chromosomal instability, NPG-high tumors are associated with lower mutational burden and neoantigens. A similar trend was observed in ER+/HER2− tumors, whereas this association was not evident in HER2+ tumors (Supplementary Fig. S2B).

### NPG Score is Associated with Less Immune Cell Infiltration and Immune Activity

**Fig. 3:**
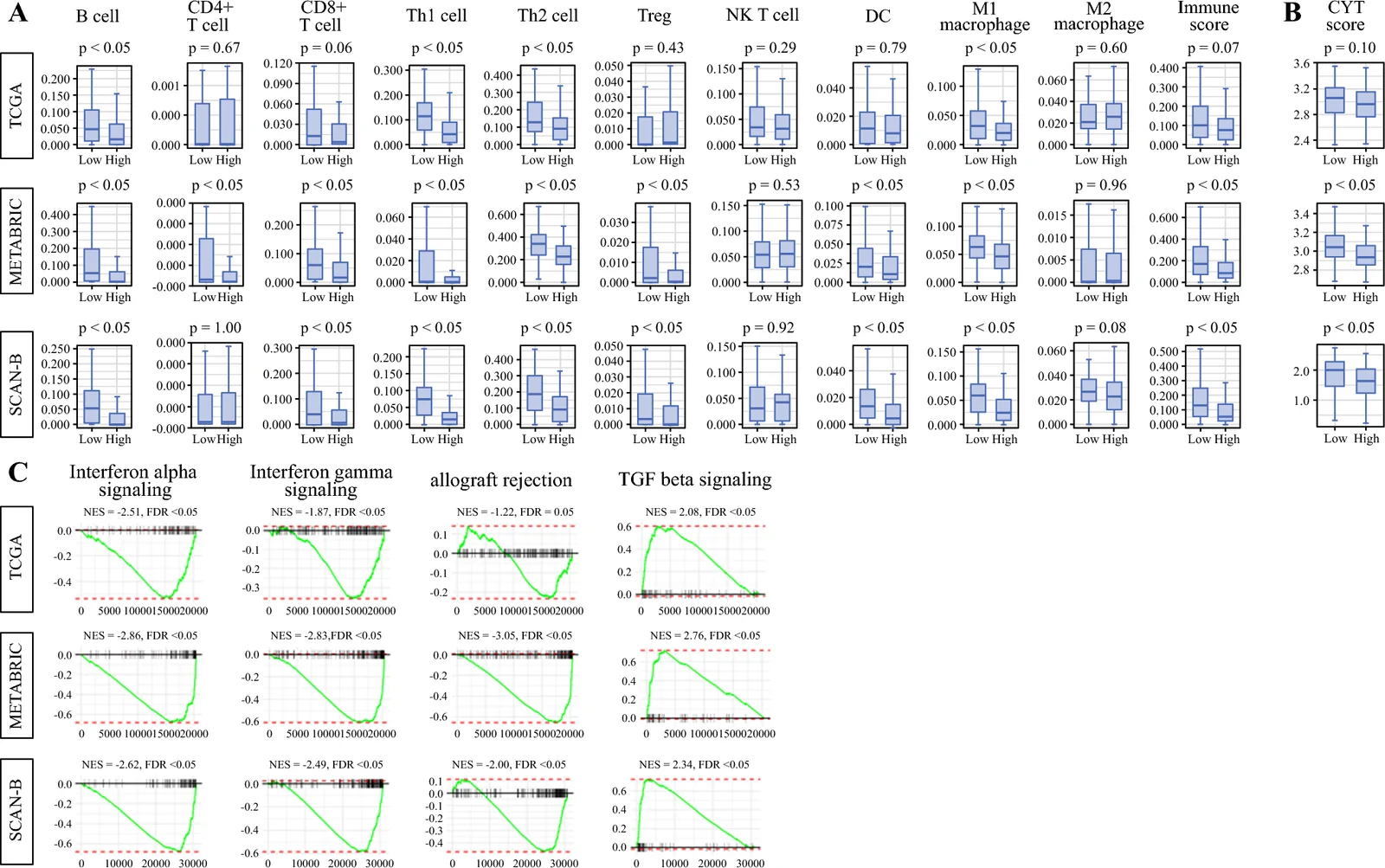
NPG score and immune cells infiltration and cytolytic activity. **A** Box plots of infiltration score of major immune cells (B cells, CD4+ T cells, CD8+ T cells, Th1 cells, Th2 cells, regulatory T cells (Treg), natural killer T cells (NK-T cells), dendritic cells (DC), M1 macrophages (M1), and M2 macrophages (M2)) calculated by xCell algorithm by NPG group in TCGA, METABRIC, and SCAN-B cohorts. **B** Box plots showing CYT score by NPG group in the three cohorts. **C** Gene set enrichment analysis (GSEA) enrichment plots for the four major immune-related Hallmark gene sets (Interferon alpha signaling, Interferon gamma signaling, allograft rejection and TGF beta signaling) in the TCGA, METABRIC, and SCAN-B cohorts. The x-axis represents ranked genes ordered by differential expression between NPG-high and NPG-low groups, and the y-axis shows the enrichment score. Normalized enrichment score (NES) and false discovery rate (FDR) are shown for each pathway

Given that NPG-high tumors were associated with lower mutational burden and SNV neoantigens, we next investigated the extent to which the NPG score was associated with differences in immune cell infiltration within the TME. Across the three cohorts, xCell algorithm-derived estimates consistently showed reduced infiltration of several immune effector cell populations in the NPG-high group, including B cells, CD8^+^ T cells, T_h_1 cells, and T_h_2 cells (all *p* < 0.05; Fig. 3A). Lower infiltration of M1 but not M2-like macrophages (*p* < 0.05 across all three cohorts) and lower dendritic cell abundance (TCGA: *p* = 0.79; METABRIC and SCAN-B: *p* < 0.05) further support the likelihood of impaired antigen presentation capacity and weakened T-cell priming, consistent with the overall reduction in immune effector cell populations. Consistent with these findings, the xCell-derived Immune score was also lower in the NPG-high group, reaching statistical significance in two of the three cohorts (METABRIC and SCAN-B: *p* < 0.05), while showing a similar but non-significant trend in TCGA (*p* = 0.07). The cytolytic activity (CYT) score, defined by the expression of GZMA and PRF1, was also significantly lower in the NPG-high group across all cohorts (all *p* < 0.05; Fig. 3B), indicating markedly diminished cytotoxic effector function in the NPG-high tumors. Consistent with these findings, GSEA revealed markedly reduced immune pathway activity in the NPG-high group. Specifically, interferon-γ response, interferon-α response, and allograft rejection pathways showed significantly less enrichment in all three cohorts (all FDR < 0.05; Fig. 3C), whereas TGF-β signaling was consistently highly enriched (all FDR < 0.05). Taken together, these results indicate that tumors with high NPG scores exhibit a less inflamed TME characterized by reduced immune effector cell infiltration and diminished immune signaling activity. Across most immune cell populations, similar patterns were observed in ER+/HER2− and HER2+ subtypes, although statistical significance was not consistently reached in all cohorts (Supplementary Figs. 3A and B).

### NPG Score is Associated with a Worse Response to Neoadjuvant Chemotherapy

**Fig. 4:**
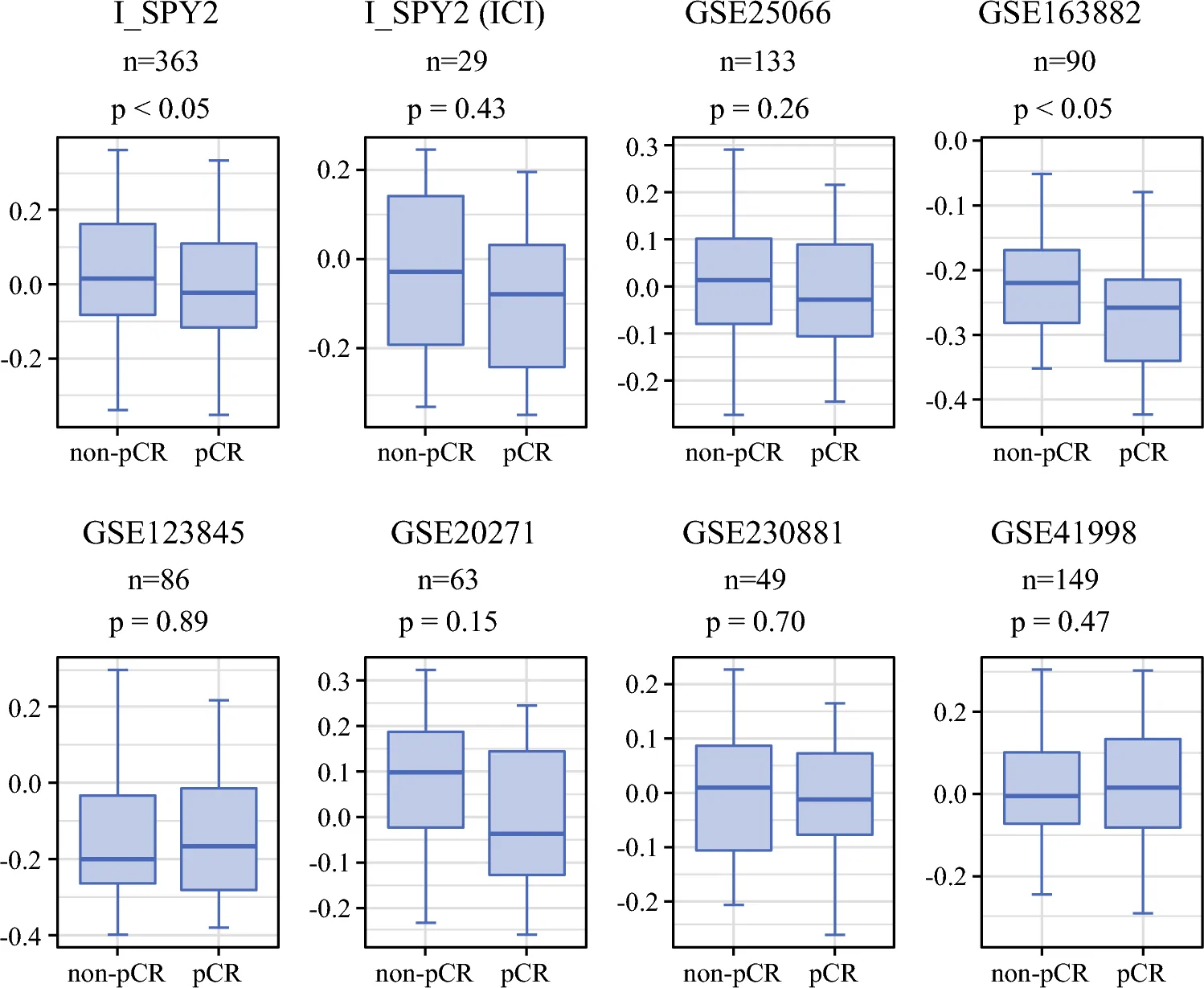
NPG scores and therapeutic response status. Box plots compare NPG scores between pCR and non-pCR patients in seven neoadjuvant chemotherapy cohorts. *P* values were calculated by using the Wilcoxon rank-sum test

It is well known that the abundance of tumor-infiltrating lymphocytes is associated with response to neoadjuvant chemotherapy (NAC).^43^ Because NPG-high tumors showed markedly less immune activity, we hypothesized that the NPG score would be associated with a worse response to NAC. To investigate this, we analyzed seven independent TNBC cohorts treated with anthracycline- and taxane-based NAC and examined the association between the NPG score and pathological complete response (pCR) after NAC. Across the seven cohorts, five demonstrated a trend toward lower NPG scores in patients who achieved pCR, although statistical significance was reached in only two cohorts (Fig. 4; ISPY2 and GSE163882: both *p* < 0.05). Similar analyses in ER+/HER2− and HER2+ subtypes did not show consistent or statistically significant associations across cohorts (Supplementary Fig. S4).

### Patients with High NPG Scores are Associated with Worse Survival

**Fig. 5:**
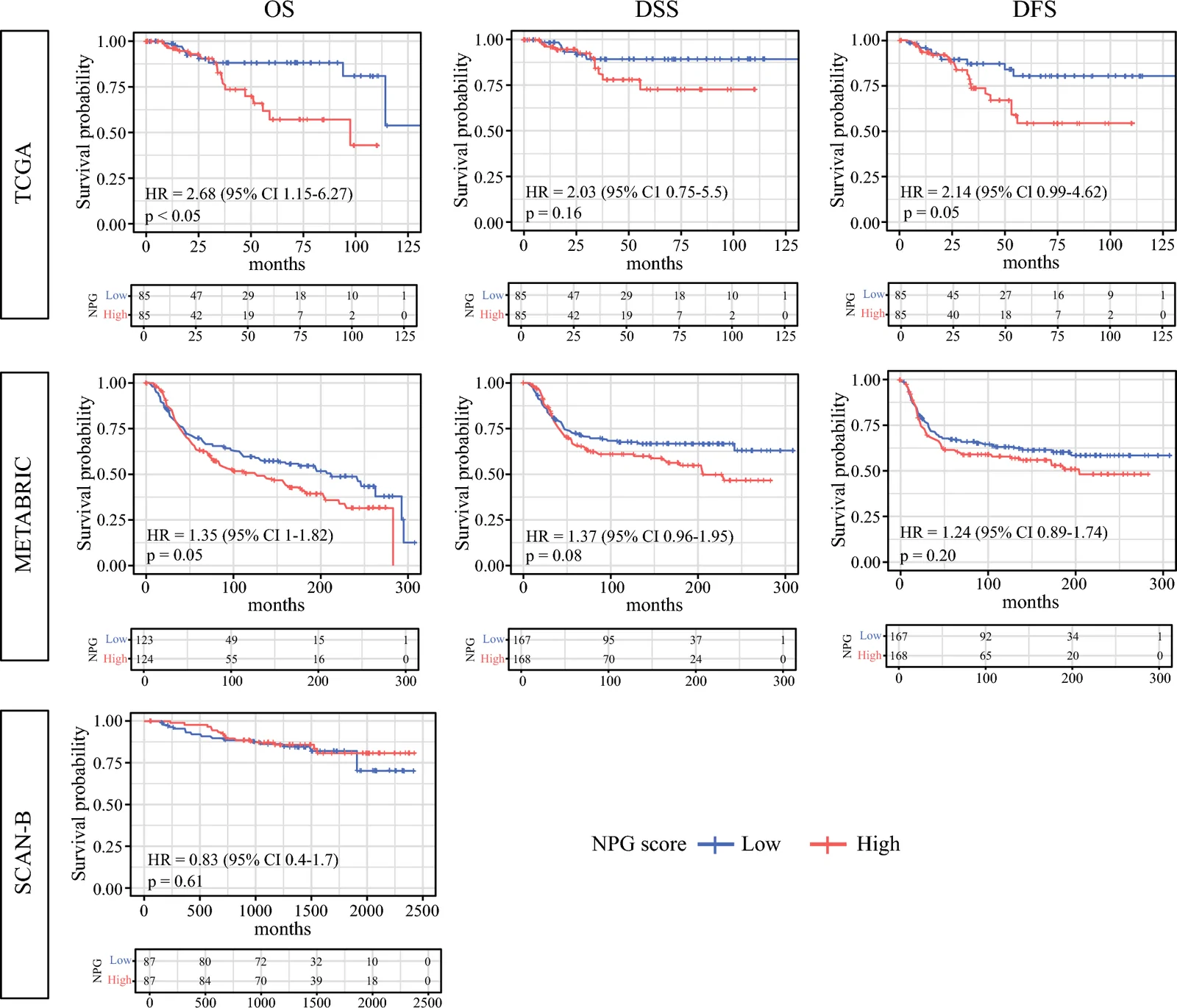
NPG score and prognostic outcomes in TCGA, METABRIC, and SCAN-B cohorts. Kaplan-Meier survival curves of overall survival (OS), disease-specific survival (DSS) and disease-free survival (DFS) by NPG score group. Red represents NPG score-high and blue represents NPG score-low group

We then investigated the association between the NPG score and survival outcome. In the TCGA cohort, high NPG scores were associated with significantly worse overall survival (OS; hazard ratio [HR] = 2.68, *p* < 0.05) with similar trends toward poorer disease-specific survival (DSS; HR = 2.03, *p* = 0.164) and disease-free survival (DFS; HR = 2.14, *p* = 0.05; Fig. 5). The METABRIC cohort demonstrated similar trends, with high NPG scores corresponding to inferior OS (HR = 1.35, *p* = 0.05), DSS (HR = 1.37, *p* = 0.08), and DFS (HR = 1.24, *p* = 0.20), although the associations did not reach statistical significance. In the SCAN-B cohort, no significant association between the NPG score and OS was observed (HR = 0.83, *p* = 0.61). No significant associations between the NPG score and survival outcomes were observed in ER+/HER2− or HER2+ subtypes (Supplementary Fig. S5).

## Discussion

This study demonstrated that the cancer-mediated process of recruiting neural projections, captured by the NPG score, identified a unique TNBC phenotype characterized by an immune-cold TME and unfavorable clinical outcomes. Across multiple large cohorts, tumors with high NPG scores unexpectedly showed profiles consistent with low cell proliferation yet exhibited reduced mutational burden, diminished immune cell infiltration, and lower cytolytic activity, indicating a profoundly suppressed anti-tumor immunity. These immune features corresponded with NPG scores associated with failure to achieve pCR after NAC across multiple cohorts. Notably, despite their lower proliferative activity, NPG-high tumors tended to experience poorer survival outcomes, suggesting that the unfavorable immune TME associated with high NPG activity may contribute to their unfavorable clinical behavior, highlighting the importance of immune-rich TME in clinical outcomes in TNBC, the most immunogenic breast cancer subtype. In addition to analyses on TNBC, ER+/HER2− and HER2+ tumors demonstrated partially overlapping but less consistent associations, suggesting that the biological impact of neural projection guidance may be particularly relevant in TNBC.

Among the various mechanisms proposed to explain the presence of neural elements within the TME, many studies have focused on the recruitment of projections from pre-existing peripheral neurons as a key contributor.^5,44^ This process is driven by neurotrophic factors released by cancer cells and directed by multiple guidance molecules that are expressed within the TME and direct neural projections toward the tumor.^6,45^ Once recruited, nerves can further influence tumor behavior through secretion of neurotransmitters and neuropeptides that affect cancer hallmarks, such as cell proliferation, invasion, metabolism, angiogenesis, and immune cell infiltration.^13,46^ Across many cancer types, the extent of such neural remodeling correlates with higher tumor histological grade, increased metastatic potential, and poorer prognosis.^2,4^ Despite its impact on tumor biology, quantifying the transcriptome of nerve fibers presents a challenge, because nuclei of neurons are not located within peripheral fibers. Most solid tumors are innervated by sensory and autonomic nerve fibers whose cell bodies reside within dorsal root ganglia or sympathetic ganglia rather than within the fiber in the bulk tumor.^3^ Only a small proportion of neuronal mRNA reaches axonal terminals through axonal transport.^47,48^ Particularly in breast cancer, where nerve fibers constitute only a small fraction of bulk tumor tissue,^15^ neither single-cell nor bulk tumor RNA-sequencing is well-suited for capturing neuronal signaling.^49^ In contrast, the molecular programs induced by new neural projections are robustly expressed in cancer cells within the TME and therefore can be reliably captured in bulk RNA-sequencing.^6^ The NPG score, derived from these pathways, showed strong associations with genes expressed in newly extending peripheral axons, supporting its validity as a surrogate measure of tumor-driven neural recruitment.

**Fig. 6:**
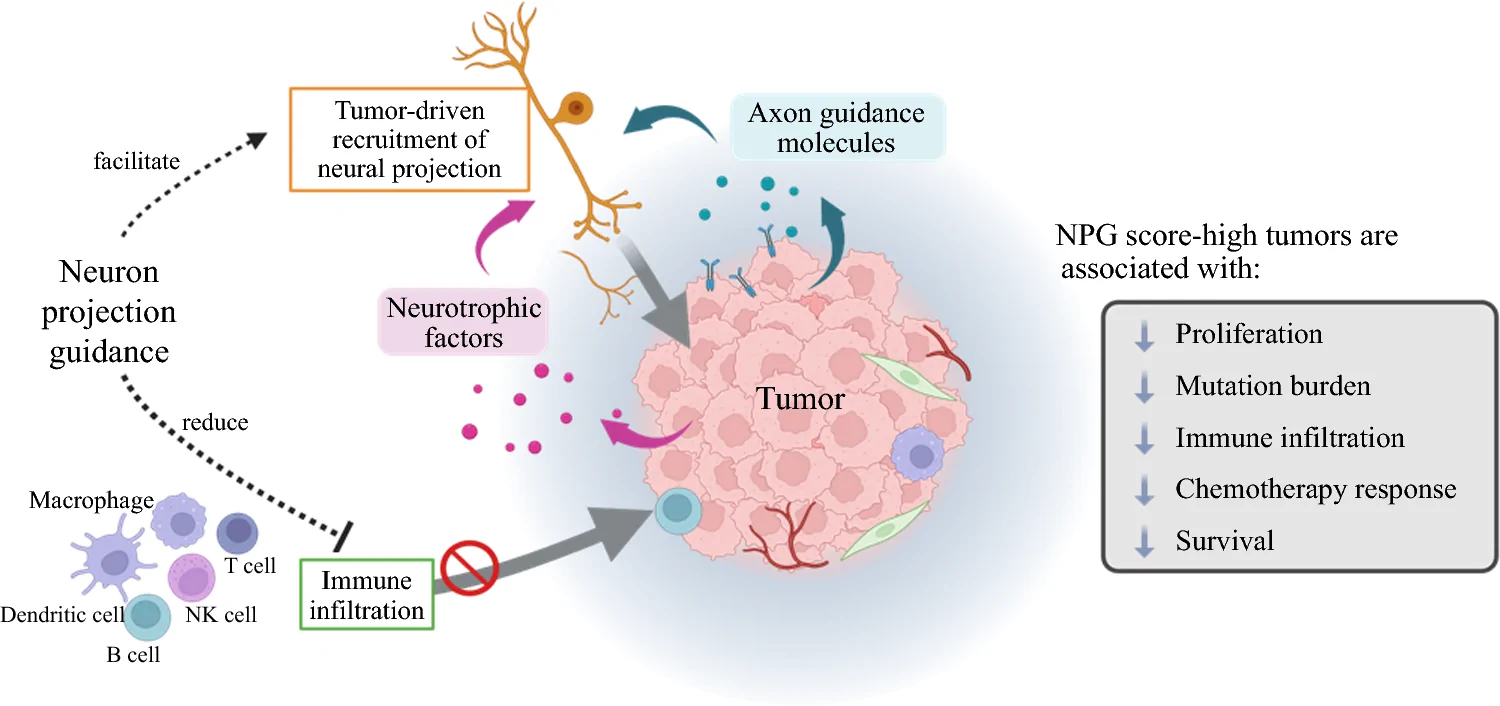
Proposed model of neural projection guidance (NPG)–associated tumor microenvironment in breast cancer. Tumor-derived neurotrophic and axon guidance signals promote neural projection toward the tumor, and NPG-high tumors are associated with an immunologically suppressed microenvironment and poorer clinical outcomes. Created with BioRender.com

Tumor-associated neural recruitment has been linked to tumor aggressiveness and poor prognosis across multiple cancers, including pancreatic, head and neck, gastric, and prostate cancers.^4,50–52^ This also has been the case in breast cancer, despite its relatively low nerve density within the TME.^53,54^ Consistent with these prior observations, tumors with high NPG scores in our study tended to show poorer clinical outcomes. However, whereas neural recruitment-associated activity is generally expected to accompany aggressive cell proliferation, NPG-high tumors exhibited lower proliferative activity, as reflected by reduced proliferation scores, lower Ki-67 expression, and less enrichment of cell proliferation-related hallmark pathways. To understand the underlying mechanisms of this unexpected pattern, we investigated the transcriptomic and immunological landscape associated with NPG score. High NPG tumors demonstrated significantly lower tumor mutational burden, including reduced nonsilent mutations and fewer neoantigens, despite showing no evidence of increased chromosomal instability. Moreover, these tumors profoundly displayed an immune-cold TME profile, characterized by reduced infiltration of immune effector populations, including CD8⁺ T cells, T_h_1 cells, T_h_2 cells, dendritic cells, M1-like macrophages, and diminished cytolytic activity. This weakened anti-tumor immune profile was further reflected in markedly decreased interferon-α or -γ responses and increased TGF-β signaling. Importantly, these immune features corresponded with high NPG scores associated with a failure to achieve pCR after NAC in multiple cohorts, suggesting that high NPG activity is linked to therapeutic resistance. Taken together, our findings indicate that the poor prognosis associated with high NPG activity is driven not by enhanced proliferative capacity but rather by a suppressed immunity and reduced treatment responsiveness, providing a new perspective on neural recruitment-related signaling in TNBC biology (Fig. 6).

Prior studies from our group, including analyses from a large randomized clinical trial, have demonstrated that higher perceived stress among breast cancer survivors is associated with worse survival outcomes.^55^ These findings highlight the need to identify intratumoral correlations of stress that may contribute to aggressive tumor biology and inform future interventional strategies. In this study, we examined neural innervation and neuron projection guidance programs as potential tumor-associated features linked to stress, with particular relevance to TNBC, a subtype characterized by aggressive behavior and limited targeted treatment options. While neuron projection guidance pathways are not currently being directly targeted, their dysregulation in TNBC may reflect underlying tumor–nerve interactions and engagement of downstream signaling networks implicated in invasion and microenvironmental remodeling. Together, these findings support neuron projection guidance–associated signatures as candidate biomarkers of stress-associated tumor biology in TNBC, warranting further investigation in translational and interventional studies.

There are several limitations in our study. First, the NPG score captures transcriptomic activity associated with neural recruitment but does not directly quantify physical nerve fibers within the TME. Second, although our analyses are based on multiple large cohorts, differences in patient composition, sequencing platforms, clinical annotations, and treatment courses may contribute to selection bias. Lastly, the observational nature of this study precludes definitive mechanistic conclusions, and experimental validation will be needed to confirm the molecular mechanisms. For instance, the mechanisms by which NPG high score TNBC suppresses immune cell infiltration, and the potential causality between cancer cell proliferation and NPG activity warrant further detailed investigation in future studies.

## Conclusions

This study highlights cancer-mediated induction of neural projection as a previously undescribed dimension of TNBC biology. Using the NPG score, we identified distinct features characterized by reduced immune activity, lower chemotherapy response, and poorer clinical outcomes, despite lower proliferative activities. These findings suggest that neural recruitment–associated signaling may potentially serve as a unique biomarker to stratify responsive from nonresponsive TNBC patients to guide therapeutic decision-making better.

## Supplementary Information

Below is the link to the electronic supplementary material.

Supplementary file1 (PPTX 17584 kb)
